# Assessment of Cyclosporine Serum Concentrations on the Incidence of Acute Graft Versus Host Disease Post Hematopoietic Stem Cell Transplantation 

**Published:** 2014

**Authors:** Sara Zeighami, Molouk Hadjibabaie, Asieh Ashouri, Amir Sarayani, Seyed Hamid Khoee, Sarah Mousavi, Mania Radfar, Ardeshir Ghavamzadeh

**Affiliations:** a *Faculty of Pharmacy, Tehran University of Medical Sciences, Tehran, Iran.*; b *Department of Clinical Pharmacy, Faculty of Pharmacy, and Research Center for Rational Use of Drugs, Tehran University of Medical Sciences, Tehran, Iran.*; c *Hematology-Oncology and Stem Cell Transplantation Research Center, Shariati Hospital, Tehran University of Medical Sciences, Tehran, Iran. *; d *Research Center for Rational Use of Drugs, Tehran University of Medical Sciences, Tehran, Iran. *

**Keywords:** Hematopoietic stem cell transplant, Graft versus host disease, Cyclosporine

## Abstract

Allogeneic hematopoietic stem cell transplantation (HSCT) is a curative treatment option for hematological disorders. Cyclosporine (CsA) is one of the major immunosuppressive agents for the prophylaxis against graft versus host disease (GvHD).

In this retrospective study, we evaluated the effects of CsA serum levels on the incidence of acute GvHD and transplant outcomes.

Retrospective study in 103 adult patients received Hematopoitic Stem Cell Transplantation (HSCT) in the Hematology-Oncology, Bone Marrow Transplantation center at Shariati Hospital in Tehran, Iran.

All participants received prophylactic regimen of cyclosporine plus methotrexate. CsA dose titration was done according to patients᾽ serum levels and drug toxicity. Serum levels tested on the twice weekly basis in first 4 weeks after transplantation.

Acute GvHD (grades II-IV) developed in 44 patients (43%, 95%CI: 33%-52%). The median time to ANC and PLT recovery was 13 days (range: 9-31 days) and 16 days (range: 0-38 days), respectively. Univariate analysis of risk factors related to aGvHD (grade II-IV) development showed a higher risk of incidence of aGvHD (grades II-IV) for patients having the lowest blood CSA concentration (<200 ng/mL) in the third weeks after transplantation (36% vs. 12%, *P *= 0.035). The only risk factors related to incidence of aGvHD grades III-IV was also blood CsA concentration at 3^rd ^week post-transplant (15% vs. 3%, *P *= 0.047). The CsA concentration at 3^rd^ week was not related to disease free survival and overall survival (*P *= 0.913 vs. *P *= 0.81) respectively.

Higher CsA serum levels in the third week post HSCT significantly decreased incidence of acute GvHD.

## Introduction

Allogeneic Hematopoietic Stem Cell Transplantation (HSCT) is considered as a curative treatment for patients with hematological malignancies like leukemia, myelodysplasia, lymphoma, and multiple myeloma. However, graft-versus-host disease (GvHD) both in its acute and chronic forms has limited the benefits of allogeneic HSCT by significant morbidity and mortality (1).

Acute GvHD which results from an interaction of donor T lymphocytes with recipient’s antigens occurs in approximately 30%-60% of patients after allogeneic HSCT (2).

Since early 1980s, Cyclosporine (CsA) in conjunction with Methotrexate (MTX) was used as a prophylactic regimen to prevent GvHD. It resulted in a substantial decrease in the incidence and severity of acute GvHD and had a remarkable influence on survival (3).

Even with newer immunosuppressive agents available in the market, CsA remains as one of the major agents in GvHD prophylaxis after allogeneic HSCT. However its dosing has been complicated by considerable intra-patient and inter-patient variability in pharmacokinetics along with its narrow therapeutic index (4). Variability in oral CsA absorption is well documented and can lead in marked differences in bioavailability of drug between different patients (5). The absolute bioavailability of oral CsA in the HSCT setting can vary between 20% and 50% (6). Different studies have shown that low CsA concentrations can result in increased risk of acute GvHD (7, 8, 9).

Such pharmacokinetic variations have been previously documented for few therapeutic agents including cyclosporine, phenytoin, and omeprazole in Iranian population (10-12). However, few studies have evaluated the variation of cyclosporine serum levels and its association with GvHD in Iranian hematopoietic stem cell transplants patients. Therefore, the aim of this retrospective study was to assess the relationship of CsA exposure and the risk of developing acute GvHD in patients receiving both reduced and conventional intensity conditioning regimens.

## Experimental

*Patients*

A total of 103 patients, aged 15-54 years, who received HSCT in the Hematology-Oncology, Bone Marrow Transplantation center at Shariati Hospital in Tehran, Iran between December 2007 and April 2010 entered our study. The study protocol was approved by the ethical committee of Tehran University of Medical Sciences and Health Services.

The primary end point of this research was to assess the relationship between the CsA serum concentrations during hospitalization period (four weeks after transplantation) to have more regular sampling and the risk of acute GvHD. Patients aged less than 15 years of age and those with poor performance status due to significant medical comorbidities were excluded from our study. The included patients received no concomitant medications known to interfere with cyclosporine pharmacokinetics. Patients, donors, and transplant characteristics are summarized in Table 1.

All patients received the conditioning regimen as inpatients in private rooms with positive-pressure laminar airflow and remained hospitalized until hematopoietic and clinical recovery. Among donors, 100 of them were HLA-identical (97 siblings, and 3 other related) and 3 were mismatched (2 sibling, and 1 other related).

The stem cell source was bone marrow in one case and peripheral blood stem cells (PBSCs) in other 102 cases.

*Preparative regimen*

The myeloablative conditioning regimen included the classical Busulfan (16 mg/Kg, oral) combined with Cyclophosphamide (120 mg/Kg, *i.v*.) in 86 patients. In three patients who had haploidentical transplantation, anti-thymocyte globulin (thymoglobulin, 10 mg/Kg, *i.v*.) was added to classical combination. All thalassemic patients received anti-thymocyte globulin (thymoglobulin), 2.5 mg/Kg on days -1 and -2 before transplantion. Thalassemic patients in class I and II received Busulfan (13.5 mg/Kg, oral) combined with Cyclophosphamide (200 mg/Kg, *i.v*.). whereas thalassemic patients in class III received Fludarabine (150 mg/m^2^, *i.v*.) instead of Cyclophosphamide in their combination regimen(FLU/BU/ATG) plus Busulfan (13.5 mg/Kg, oral).

**Table 1:** Patients, Donors and Transplant characteristics

**Group**	**Total**
Number Patients	103
Median age, year (range)	26 (15-54)
Median BMI, kg/m2 (range)	22.6 (15-36.8)
Sex	
Male	70 (68)
Female	33 (32)
Diagnosis	
CML	5 (5)
ALL	34 (33)
AML	50 (49)
Thalassemia	14 (13)
Status at tx	
CR1	59 (57)
CR2	18 (18)
Other (CR3/PIF/PR/relapse refractory)	8 (7)
Not applicable (CML/Thalassemia)	18 (18)
Conditioning regimen	
Bu/Cy	86 (84)
Bu/Cy/ATG	12 (12)
Flu/Bu/ATG	5 (5)
Median time to transplantation, month (range)	9 (<1-332)
**Donors**	
Median age, year (range)	24 (6-67)
Sex	
Male	60 (58)
Female	43 (42)
Sex-mismatch	46 (45)
Female donor to male recipient	28 (27)
Donor type	
Matched related donor	97 (94)
Matched unrelated donor	6 (6)
ABO-mismatch	32 (31)
CMV serology antibody*	
R+/D+	98 (95)
R-/D+	1 (1)
R+/D-	4 (4)
Median TNC infused×108 /kg (range)	9.74 (5.56-18.24)
Median Total MNC infused×108 /kg (range)	7.92 (4.89-11.99)
Median CD34+ cells infused (range)*	3.61 (.49-32.3)
Median CD3+ cells infused (range)*	271.5 (34-547)
Median follow up time, month (range)	14 (1-37)

All patients had peripheral blood stem cell transplantation and received CsA+MTX as GVHD prophylaxis.

^∗^ CD34+ and CD3+ were available for 88% (91) of patients.

BMI indicates body mass index; CML, chronic myeloid leukemia; ALL, acute lymphoblastic leukemia; AML, acute myelogenous leukemia; tx, transplantation; CR, complete remission; PIF, primary induction failure; PR, partial remission; BU, busulfan; CYC, cyclophosphamide; ATG, antithymocyte globulin; FLU, fludarabine; ABO, blood type; CMV, cytomegalovirus; R, recipient; D, donor; WBC, white blood cells; MNC, mononuclear cells; (): percent; CsA, cyclosporine; MTX, methotrexate; (): percent.

*GvHD prophylaxis*

Acute GvHD was diagnosed by clinical manifestation and graded based on standard criteria (13).

All patients received CsA and short course of Methotrexate (MTX). Based on the ward protocol and pervious experiment of CsA toxicities on our patients, Intravenous CsA started as 1.5 mg/Kg/day in two divided doses as two-hour infusion two days before graft infusion and then increased to 3 mg/Kg/day from day +7 post-transplant(dosing regimen is based on our hospital protocol). It was switched to oral CsA (8 mg/Kg/day) according to patients᾽ mucositis condition and ability of taking oral medications. CsA was usually continued for 180 days and finally tapered and discontinued in the next 6 months.

CsA dose was adjusted based on drug trough levels (100-300 ng/mL) and toxic side effects like nephrotoxicity. MTX was administered at a dose of 10 mg/m^2^ on day +1 and then 6mg/m^2^ on days +3, +6, and +11 post-transplant. Folinic acid15 mg/m^2^/day was administered 24 hours after MTX dose in order to minimize myelosuppression and mucosal damages.

*Supportive care*

Supportive measures like hydration, antibacterial, antiviral and antifungal prophylaxis, blood products and total parenteral nutrition were provided according to clinical standard procedures.

Hematopoietic recovery was defined as absolute neutrophil count (ANC) count greater than 0.5×10^9^/L and platelet count greater than 20×10^9^/L without platelet transfusion for 3 consecutive days. Disease free survival (DFS) was defined as survival in continuous disease free period without any event. Overall survival (OS) was defined as survival with or without relapse.

*Drug analysis*

CsA levels were measured in EDTA-anticoagulated whole blood using Immunotechcyclosporine direct radioimmunoassay (Beckman Coulter, Prague, Czech Republic). The method quantitates 5-2000 ng/mL CsA with coefficient of less than 5.8% and 8% for intra- and inter-assay variations, respectively. The range of trough level between 100-300 ng/mL was considered therapeutic.

*Statistical analysis*

Patients, donors and transplant characteristics were compared between groups using chi square statistics for discrete variables and Mann-Whitney test for continuous variables. Probabilities of neutrophil and platelet (PLT) recovery, incidence of acute and chronic GvHD, OS and DFS were estimated by the Kaplan Meier method and compared between groups (CsA concentration group) by the log rank test in a univariate analysis. Cox proportional hazard regression model (with backward stepwise method) was used for identifying independently effect of blood CsA concentration adjusting of other potential risk factors (Potential risk factors that were studied are listed in Table 1). In the performing of regression models, collinearity among the covariates was checked. In all analysis the median normal blood CsA concentration (200 ng/mL) was used to categorize patients with low and high blood CsA concentrations.

## Results

One hundred three consecutive patients with AML (49%), ALL (33%), CML (5%) and Thalassemia (13%), who had the inclusion criteria were enrolled in the study. The median time to ANC and PLT recovery was 13 days (range: 9-31 days) and 16 days (range: 0-38 days), respectively (Table 2).

**Figure 1:**
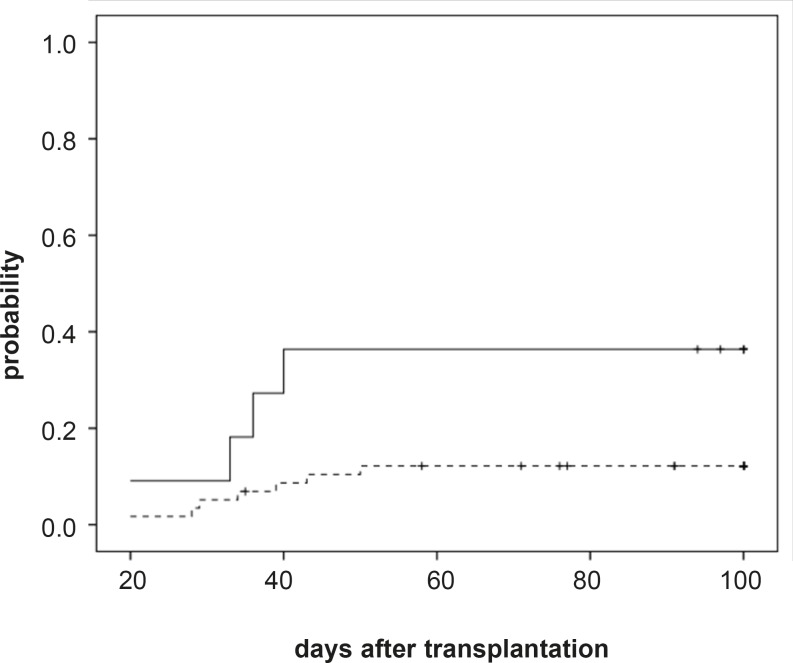
Incidence of acute GvHD grades II-IV after transplantation by CsA concentration level.

The median concentrations of CSA in the blood at 1, 2, 3, and 4 weeks after transplantation were 107 (range: 40-384), 209 (range: 61- 690), 325 (range: 53-871), and 437 (range: 140-780) ng/mL, respectively (Table 2, Figure 1).

Acute GvHD grades III-IV developed in 22 patients (21%, 95%CI: 13%-28%). Only risk factors related to incidence of aGVHD grades III-IV was blood CsA concentration at 3^rd^ week and there was no relations between incidences of severe aGvHD and factors listed in Table 1 or blood CsA concentration in other weeks. The incidence of grades III-IV acute GvHD after 3^rd^ week of transplantation, was 15% (2/13) vs. 3% (2/70) in the patients with CsA concentration lower than 200 ng/mL vs. patients with CsA concentration equal or higher than 200 ng/mL (*P*=.047).

**Table 2:** Engraftment, Transplantation and GvHD

Median days to tx, (range)	8 (5-34)
Median days of hospitalization (range)^a^	17 (13-35)
Median days to	
ANC more than 0.5×10^9^/L	13 (9-31)
PLT more than 20×10^9^/L	16 (0-38)
Median transfused RBC unit (range)	3 (1-13)
No. of patients without transfused RBC	43 (42)
Acute GvHD	
None	29 (28)
Grade I	30 (29)
Grade II	22 (21)
Grade III	18 (18)
Grade IV	4 (4)
Median CsA concentration, ng/mL (range)	
First week (n=101)	106.5 (40-384)
Second week (n=99)	209 (61-690)
3rd week (n=101)	325 (53-871)
4th week (n=50)	436.5 (140-780)
No. of pts with CsA concentration > 200 ng/mL^b^	
First week (n=101)	8 (8)
Second week (n=99)	54 (55)
3rd week (n=101)	83 (82)
4th week (n=50)	47 (94)
Relapse/Recurrence	16 (16)
Survival	
Alive	76 (74)
Death	27 (26)

^a^ Period of hospitalization calculate from transplantation.

^b^ Percent was calculated in no. of pts with known CsA level.

Tx indicates transplantation; ANC, absolute neutrophil count; PLT, platelet; RBC, red blood cell; GvHD, graft versus host disease; (): percent.

Acute GvHD (grades II-IV) developed in 44 patients (43%, 95%CI: 33%-52%) with median 11 (range: 6-50) days after transplantation. Univariate analysis of risk factors related to aGVHD (grade II-IV) development showed a higher risk of incidence of aGVHD (grades II-IV) for patients having the lowest blood CSA concentration in the third weeks after transplantation (36% (4/11) vs. 12% (7/58), *P*=.035, Figure 1). However RBC transfusion showed a relation with incidence of aGvHD grades II-IV in univariate analysis but in the multivariate analysis it was not significant (*P*=.419). In the multivariate analysis we found that higher CsA concentration (CsA concentration more than 200 ng/mL) during the third week following graft infusion and before onset of aGvHD, was the significantly associated with a reduced risk of grade II-IV aGVHD (*P*=..048, RR: 3.46; 95% CI: 1.01-11.90). Because of few sample size, multivariate analysis wasn’t performed for severe grade III-IV aGVHD.

The median follow-up time of surviving patients was 14 (range: 1-37) months after transplantation and the OS and DFS at one year after transplantation was 74% (Se: 5%), 67% (Se: 5%), respectively. Causes of death based on CsA concentration at 3^rd^ week are shown in Table 3. The CsA concentration at 3^rd^ week was not related to DFS and OS (*P*=.913 and *P*=.813, respectively). The risk factor related to OS and DFS was status at transplantation and patients in the first complete remission has higher OS and DFS probability (*P*=.008 and *P*<.001, for OS and DFS respectively). The relative risk of relapse or death for patients in first complete remission status at transplantation was .27 (95% CI: .139-.537, *P*<.001). 

**Table 3:** Causes of Death.

Cause	Total	CsA concentration at 3^rd^ week, ng/mL	
		<200	>=200
Graft rejection or failure	11	2	9
GvHD	8	2	6
Infection	4	--	4
New malignancy	1	--	1
Pleural effusion	1	--	1
DIC	1	--	1
unknown	1	--	1
Total	27	4	23

GvHD indicates graft versus host disease; DIC, Disseminated Intravascular Coagulation.

The relative risk of death for patients in first complete remission status at transplantation was .35 (95% CI: .159-.759, *P=*.008).

## Discussion

Cyclosporine was first introduced in late 1970s (14) and has become an important agent as an immunosuppressant in the setting of different transplant (renal, hepatic, and cardiac) as well as HSCT (15). In the early 1980s, CsA trough level monitoring was introduced into clinical practice for the first time and despite its widespread use, there have been controversies over the most appropriate way of performing drug monitoring of CsA. A number of studies demonstrated that C2 (CsA serum level 2 hours post dose) monitoring in solid organ transplantation could have superior outcomes in terms of rejection and toxicity profile compared to trough level monitoring (16, 17). In 1988, the Seattle group showed that low CsA levels can result in increased risk of aGvHD, and the levels should be monitored in recipients of bone marrow transplant (7). However, CsA pharmacokinetics is highly variable. Factors like transplant type, patient age and concurrent drug therapy can influence serum concentrations of the drug (18). 

There are studies demonstrating an inverse relation between trough CsA concentrations and incidence of GvHD (19, 20). Martin *et al*. evaluated the relationship between CsA trough blood concentrations and incidence of aGVHD in pediatric patients under HSCT and found that there was a strong relationship between trough blood concentrations during the first two weeks after transplantation and the severity of aGVHD in these patients (8). Similarly, Mallard *et al*. in a retrospective study revealed that patients who had the lowest concentration of CsA in the first and second weeks after allogeneic HSCT experienced a significantly higher risk of severe aGVHD (20). However, there are a few trials failed to show a significant relationship between trough levels and acute GvHD, also conflicting data regarding CsA levels and the risk of nephrotoxicity (21-23). In a Japanese study by Kurokawa *et al*., maintaining higher levels of CsA (around 500 ng/mL) with continuous intravenous infusion significantly decreased the incidence of both acute and chronic GvHD (24).

The major finding in this current study was that maintaining CsA trough levels equal or greater than 200 ng/mL in the third week resulted in lower incidence of aGvHD in the following weeks. Similar results were found in other studies where lower serum levels of CsA during the 3rd week resulted in higher incidence of aGvHD (25, 26). These findings could be due to the median time of donor leukocyte engraftment around day 14 which causes development of aGvHD during the 3^rd ^week post HSCT.

## Conclusion

We found significant benefits of maintaining higher serum concentration of CsA during the 3^rd^ week post HSCT in minimization of aGvHD. However further prospective clinical trials are necessitated to evaluate the significance of CsA levels and its relationship with development of aGvHD which have a significant impact on transplant outcomes.
